# Progress in the Study of the Effects of Exercise on Affective and Anxiety Disorders

**DOI:** 10.3389/fpsyt.2014.00153

**Published:** 2014-11-05

**Authors:** Felipe Barreto Schuch

**Affiliations:** ^1^Hospital de Clínicas de Porto Alegre, Porto Alegre, Brazil

**Keywords:** depression, anxiety, bipolar, quality of life, BDNF, galanin, meditative movement, genetic marker

Exercise has received great attention as a treatment for affective and anxiety disorders, and several studies have highlighted its mental and physical health benefits for these populations. Despite the innumerous benefits, however, there are many issues in the literature that need further exploration.

In depression, exercise appears to moderately improve depressive symptoms. Blake (1) and Deslandes (2) reviewed the literature pointing to recent findings about the current use and efficacy of exercise in depression, and the challenges in treating depression with exercise. Some of these points, as the efficacy and effectiveness of exercise, and some potential factors related to its efficacy and effectiveness, were revisited in Schuch and Fleck (3). This paper highlighted the potential implications of the heterogeneity of depression diagnosis, the psychometric instruments, and other non-specific factors on the response rates found in clinical trials. In the same line, Stanton et al. (4) reviewed the effects of exercise, analyzing the guidelines that have discussed the prescription of exercise in major depression, bipolar disorder, and post-natal depression. Still related to prescription of exercise, Paine and Crane-Goodreau (5) reviewed studies using meditative movements on the treatment of depression and anxiety, suggesting its potential role in the treatment of depression and anxiety.

Quality of life (QoL) improvement is a major challenge in the depression treatment. Reinforcing the discussion of Blake (1) regarding QoL, a longitudinal study enrolling more than 15,000 participants showed that recreational activity improved some of the negative impact of depression on health-related QoL (6).

The mechanisms related to the antidepressant and anxiolytic effects of exercise remain unclear, though the literature reveals some insights in this regard. Anderson and Shivakumar (7) provided a review of the several potential physiological (hypothalamic–pituitary–adrenal axis, monoamines, opioids, and neurotrophic) and psychological (anxiety sensitivity and exposure, self-efficacy, and distraction) explanations to the anxiolytic effects of exercise. Similarly, Deslandes (2) discussed the potential role of brain-derived neurotrophic factor (BDNF) and neurogenesis in the antidepressant effects of exercise. Additionally, Holmes (8) analyzed the influence of Galanin and the interaction between Galanin and BDNF in the role of exercise-induced stress resilience. Genetic mechanisms, as pleiotropy, provide a possible explanation for some depressed populations’ lack of response to exercise, as well as the association between inactivity and depression (9). The neuroimmune system appears to be implicated in the pathophysiology of depression. Meanwhile, exercise has shown effects on several immunological biomarkers. In this regard, Eyre et al. (10) provide an extensive review regarding some specific factors such as changes on some factors as interleukins (1 and 6), macrophage migration inhibitory factor, central nervous system-specific autoreactive CD4+ T cells, M2 microglia, quiescent astrocytes, CX3CL1, and insulin-like growth 35 factor-1, Th1/Th2 balance, pro-inflammatory cytokines, C-reactive protein, M1 microglia, and reactive astrocytes.

The topic presented several discussions regarding the current literature, the limitations of present studies, as well as several potential biological mediators of the relationship between exercise and depression/anxiety. The discussion may help researchers and other professionals of mental health form a broader comprehension of the exercise–depression relationship.

## Conflict of Interest Statement

The author declares that the research was conducted in the absence of any commercial or financial relationships that could be construed as a potential conflict of interest.
